# A System of Practical Medicine

**Published:** 1886-07

**Authors:** 


### Book Reviews.

A System of Practical Medicine by American Authors. Edited by Wm. Pepper, M. D., etc., assisted by Louis Starr, M. D., etc. Vol. IV., Diseases of the Genito-Urinary and Cutaneous Systems, Medical Ophthalmology and Otology. Philadelphia: Lea Brothers & Co. 1886.

This volume opens with chapters upon Diseases of the Kidneys, including the Pelvis of the Kidneys, by Robert T. Edes, and upon Diseases of the Parenchyma of the Kidneys and Perinephritis, by Francis Delafield. The high reputation of these authors as clinicians and pathologists is a guarantee of the excellence of their work.

The chapter upon Hæmaturia and Hæmoglobinuria or Hæmatinuria, by James Tyson, is devoted chiefly to the consideration of malarial hæmaturia, a subject of special interest to practitioners in the South, both from the more frequent occurrence of the disease in this section and from the recent valuable studies of the subject by members of the profession in this State. The author proves by a case in his own experience that negroes are not exempt from this disease, as has been claimed. The treatment recommended consists of large doses of quinine (16 to 20 grains), which the author regards as giving "brilliant and satisfactory results." This view is not altogether in accord with the experience of Southern practitioners. It is indisputable that cases sometimes occur in which all modes of treatment are equally unsatisfactory, as in a typical case reported by L. E. Borcheim, of Atlanta. (*Medical Record*, 1882, Vol. XXII., p. 699.)

Chapters upon Chyluria, also by James Tyson, Diseases of the

Bladder, by Edward L. Keyes, and Seminal Incontinence, by Samuel W. Gross, complete this portion of the volume, and are followed by the section upon subjects pertaining to gynæcology.

An article upon Displacements of the Uterus, by E. C. Dudley, presents a marked advance upon the teachings of the text-books heretofore in use. It wisely rejects the theory, of which Grailly Hewitt is the most prominent exponent, that displacements are diseases *per se*, and as such are productive of evil consequences. The author strikes the true key-note in the pathology and treatment of these conditions when he says: "The application of the pessary is based upon the general proposition that if the cervix is normally placed, the body of the uterus will, in the absence of complications, take care of itself." He teaches that the symptoms which accompany flexions and versions are due to prolapse of the organ and the treatment recommended is based upon this principle. These teachings are not new. The same views will be found in an article entitled "The Use and Abuse of Pessaries," published in the ATLANTA MEDICAL AND SURGICAL JOURNAL for December, 1885. It is gratifying to observe that the author discards the time-honored notion that pressure of the uterus upon the bladder produces irritation of that viscus, and attributes that symptom to its true cause—traction upon the vesical neck. But, on the other hand, it is surprising to notice that no mention is made of the genu-pectoral position in the treatment of retroflexions.

The article by Reeve, upon Disorders of the Uterine Function, treats very satisfactorily and thoroughly of amenorrhœa, dysmenorrhœa and menorrhagia. Its practical value is in nowise impaired by the dogmatic statement of the author that menstruation "is most intimately connected with and dependent upon ovulation, each menstrual discharge being the sign and evidence of the maturation and expulsion of one ovum or more." This positive assertion is not in accord with the results of the latest investigations. A very recent and complete study of the subject by Feoktistow (Archiv. f. Gyn., Bd. XXVII, Cah. 3) leads to the opposite conclusion, thus corroborating the results of the extensive researches of Sinéty, Haussman and Leopold.

The excellent article by Baer, upon Inflammation of the Pelvic Cellular Tissue and Pelvic Peritoneum, is marred by his adoption of the nomenclature of Matthews Duncan. Parametritis and perimetritis are awkward and unscientific terms—awkward, because their similarity is liable to create confusion, especially in the mind of the novice, and unscientific, because they fail to define the tissue in which the lesion is located, and imply a relation to the uterus, which is by no means invariable or essential. Pelvic cellulitis and pelvic peritonitis are much more simple and expressive terms.

The section upon Pelvic Hæmatocele, by Thomas, is essentially a reproduction of the chapter upon that subject in the last edition of his work on Diseases of Women. The only points of difference are the addition of pelvic peritonitis and ectopic gestation to the list of causes, and the recommendation of abdominal incision in those cases in which, suppurative action having occurred, the abscess points toward the abdominal walls.

Fibrous Tumors of the Uterus, Sarcoma of the Uterus, and Carcinoma of the Uterus, are treated of by Wm. H. Byford. In the palliative treatment of cases of cancer of the womb which do not admit of operation, the author relies chiefly upon removal of the necrosed tissue by the curette, a painful, bloody and somewhat dangerous operation. A recent article by Sarraute (Nouvelles Archives d'Obst. et de Gyn., Vol. I., No. 3) shows that much more satisfactory results in the relief of pain, the suppression of hemorrhage, the prevention of septic intoxication and the limitation of the spread of the disease may be accomplished by appropriate local medication.

The chapter upon Diseases of the Ovaries and Oviducts, by Wm. Goodell, is admirable in all respects. It treats of an important class of subjects with thoroughness and lucidity, and throws light upon many details which, to the inexperienced surgeon, are frequently obscure and uncertain. The attractive style which has characterized the previous writings of this author suffers no diminution in this excellent monograph. But the reader will probably regret, in view of the present importance and prominence of the subject, that but a single page is devoted to the consideration of cysts of the oviducts.

Alexander J. C. Skene is a recognized authority upon Diseases of the Urinary Organs in Women. Any article upon that subject from his pen might therefore be expected to embody the latest advances in the treatment of this class of affections. The failure to make any mention of Emmet's button-hole operation constitutes an unpardonable defect in what is otherwise an excellent treatise.

To those American readers who have been accustomed to pin their faith to the theories of Emmet, and who have not familiarized themselves with the tenets of the German school of gynæcology, the views enunciated by W. W. Jaggard upon Acute and Chronic Metritis will possess at least the charm of novelty. The medical mind in this country is so thoroughly saturated with the teachings which emanate from the New York State Woman's Hospital, that it will be difficult to convince the average American physician that acute metritis may exist outside of the puerperal state; that chronic metritis is not a misnomer for subinvolution; that it frequently exists without the presence of laceration of the cervix, and that local blood-letting and amputation of the collum uteri are among the most valuable resources in its treatment.

Yet such is the teaching of the German school which forms the basis of this article. The curious reader may compare the one with the other and make his choice of authorities.

Diseases of Pregnancy and of the Menopause, by W. W. Jaggard, and Abortion, by George J. Engelmann, are treated at a length somewhat disproportionate to the amount of space allotted to other equally important subjects. In the treatment of the vomiting of pregnancy, nothing new is offered, and no notice is given to the valuable article upon the subject read by Dr. Henry F. Campbell at the last meeting of the American Gynæcological Society. (Trans. Amer. Gyn. Society, Vol. X.) The results in the cases therein cited, which were treated by the genu-pectoral posture, are such as to merit the most earnest attention at the hands of the profession.

The remainder of the volume is devoted to Diseases of the Muscular System, by J. C Wilson, Jas. Tyson and Mary Putnam Jacobi; Diseases of the Skin, by Louis A. Duhring and Henry W. Stelwagon; Medical Ophthalmology, by Wm. F. Norris, and Medical Otology, by George Strawbridge.

The volume is well gotten up and reflects great credit upon the publishers. The mechanical execution is all that could be desired, and the freedom from typographical errors is somewhat remarkable for a first edition. V. O. H.

